# Correlation of umbilical cord blood hormones and growth factors with stem cell potential: implications for the prenatal origin of breast cancer hypothesis

**DOI:** 10.1186/bcr1674

**Published:** 2007-05-14

**Authors:** Todd M Savarese, William C Strohsnitter, Hoi Pang Low, Qin Liu, Inkyung Baik, William Okulicz, David P Chelmow, Pagona Lagiou, Peter J Quesenberry, Kenneth L Noller, Chung-Cheng Hsieh

**Affiliations:** 1 Department of Cancer Biology, University of Massachusetts Medical School, 364 Plantation Street, Worcester, MA 01605, USA; 2 Department of Obstetrics and Gynecology, Tufts-New England Medical Center, 750 Washington Street, Boston, MA 02111, USA; 3 Department of Neurology, University of Massachusetts Medical School, 55 Lake Avenue North, Worcester, MA 01655, USA; 4 Department of Physiology, ILAT Steroid RIA Laboratory, University of Massachusetts Medical School, 55 Lake Avenue North, Worcester, MA 01655, USA; 5 Department of Hygiene and Epidemiology, University of Athens Medical School, 75 M. Asias Street, Goudi, GR-115 27, Athens, Greece; 6 Division of Hematology/Oncology, Rhode Island Hospital, 593 Eddy Street, Providence, RI 02903, USA

## Abstract

**Introduction:**

Prenatal levels of mitogens may influence the lifetime breast cancer risk by driving stem cell proliferation and increasing the number of target cells, and thereby increasing the chance of mutation events that initiate oncogenesis. We examined in umbilical cord blood the correlation of potential breast epithelial mitogens, including hormones and growth factors, with hematopoietic stem cell concentrations serving as surrogates of overall stem cell potential.

**Methods:**

We analyzed cord blood samples from 289 deliveries. Levels of hormones and growth factors were correlated with concentrations of stem cell and progenitor populations (CD34^+ ^cells, CD34^+^CD38^- ^cells, CD34^+^c-kit^+ ^cells, and granulocyte–macrophage colony-forming units). Changes in stem cell concentration associated with each standard deviation change in mitogens and the associated 95% confidence intervals were calculated from multiple regression analysis.

**Results:**

Cord blood plasma levels of insulin-like growth factor-1 (IGF-1) were strongly correlated with all the hematopoietic stem and progenitor concentrations examined (one standard-deviation increase in IGF-1 being associated with a 15–19% increase in stem/progenitor concentrations, all *P *< 0.02). Estriol and insulin-like growth factor binding protein-3 levels were positively and significantly correlated with some of these cell populations. Sex hormone-binding globulin levels were negatively correlated with these stem/progenitor pools. These relationships were stronger in Caucasians and Hispanics and were weaker or not present in Asian-Americans and African-Americans.

**Conclusion:**

Our data support the concept that *in utero *mitogens may drive the expansion of stem cell populations. The correlations with IGF-1 and estrogen are noteworthy, as both are crucial for mammary gland development.

## Introduction

It has been hypothesized that the *in utero *environment and perinatal factors may influence breast cancer risk of the offspring later in life [1]. Epidemiological and experimental data have provided support to this hypothesis using surrogate indicators of the *in utero *and perinatal environment. Birth weight has thus been positively correlated with lifetime risk of breast cancer [2], as well as other cancers [3-5]; in addition, the offspring of preeclamptic pregnancies, probably reflecting an abnormal endocrine environment, have a markedly reduced lifetime risk of breast cancer [6].

A stem cell burden theory has been invoked to explain how *in utero *and perinatal factors might impact lifetime breast cancer risk [7-10]. The tenets of this theory include the following. Firstly, the breast cancer risk is related to the pool size of breast stem cells, which may be determined *in utero *or early in life. A second tenet is that individuals with relatively elevated *in utero*/perinatal levels of growth factors (for example, insulin-like growth factor-1 (IGF-1)) and hormones (for example, estrogens) that act as mammary epithelial cell mitogens will have relatively large and mitotically active pools of breast stem cells. This would increase the probability that oncogenic mutations will occur in one of these cells. Thirdly, in the presence of elevated levels of mitogens, such individuals might also have a general increase in various stem cell pools and possibly birth weights. When first proposed, this concept was highly speculative [1,7]. Since that first proposal, however, the existence of stem-like, multipotential breast epithelial cells in both mice [11,12] and humans [13,14] has been established. Additionally, there is evidence for malignant breast 'stem' cells with some properties of normal breast stem/progenitor cells, suggesting that the former may be derived from the latter [15].

Preliminary and indirect support for this stem cell-based hypothesis came from a pilot study on 40 umbilical cord blood samples from infants delivered in the Worcester, MA, area [16]. In that study, cord blood plasma levels of several key sex hormones, including estradiol, estriol, testosterone and progesterone, the sex hormone-binding globulin (SHBG) and certain growth factors including prolactin and IGF-1, as well as one of the major IGF-1 binding proteins, insulin-like growth factor binding protein-3 (IGFBP-3), were assayed to determine whether they correlated with the density of cord blood-derived hematopoietic stem cell and progenitor cell populations, serving as surrogates for overall stem cell potential. Such populations included cord blood CD34^+ ^cells, representing progenitors of hematopoietic cells, endothelial cells and possibly other cell types, CD34^+^CD38^- ^cells, representing primitive hematopoietic stem/progenitor cells, and granulocyte–macrophage colony-forming units (GM-CFU), representing a functional measure of multipotential, proliferative hematopoietic precursor cells [17]. There was indication that cord blood plasma levels of IGF-1, and to a lesser extent estriol and testosterone, are positively correlated with the density of cord blood CD34^+^cells, CD34^+^CD38^- ^cells, and GM-CFU [16].

The current study was carried out to determine whether the correlations between cord blood growth factors and hormone levels and stem cell populations are robust in a larger, more broadly based, population sampling. A total of 289 cord blood samples incorporating 40 samples from the pilot study and 249 samples from the Tufts–New England Medical Center, covering a more racially/ethnically diverse area, were analyzed.

## Patients and methods

### Study subjects

Study subjects were recruited from one of two sources: participants in the Worcester, MA-based American Red Cross cord blood program, in which hematopoietic stem cells from umbilical cord blood were collected for transplantation; and pregnant women delivering at the Tufts–New England Medical Center in Boston, MA. In all instances, eligible participants were 18 years of age or older, bearing a single fetus with no anomalies by sonography and with no indication of umbilical cord prolapse or amniotic fluid embolism, and were negative for human immunodeficiency, hepatitis B, and hepatitis C viruses. Each participant signed a consent form approved by the human subjects committee of the institution where deliveries occurred.

Umbilical cord blood samples were obtained from full-term (gestational age ≥37 weeks) infants. Prior to collection, the cord was cleaned with alcohol and Betadine. Umbilical cord blood was drained from the umbilical vein using a 16-gauge needle and was collected in a collection bag containing 35 ml citrate–phosphate–dextrose anticoagulant (Baxter Health Care, Deerfield, IL, USA). Samples were generally obtained with the placenta *in utero*; however, cord blood was collected after delivery of the placenta in the case of childbirths requiring cesarean section.

Women consenting to the study were asked to provide their age, race, education, number of prior pregnancies and live births, pregnancy complications, and family history. Information on the birth weight and gestational age of the infant was also recorded. The study protocol was approved by the institutional review boards of the American Red Cross, the University of Massachusetts Medical School, the University of Massachusetts/Memorial Health Care System, St Vincent's Hospital, and the Tufts–New England Medical Center.

### Processing of umbilical cord blood samples

Cord blood samples were kept at room temperature after collection, during transportation, and at the initial processing, always within 24 hours of the time of collection. The total volume of the cord blood samples was measured, and a complete blood count with automated differential was performed on an aliquot of the blood using a model A^C^T-diff Coulter Counter (Beckman Coulter, Hialeah, FL, USA). Each cord blood sample was centrifuged for 30 min at 400 × *g *in a Sorvall Legend RT (Kendro Laboratory Products, Osterode, Germany centrifuge at room temperature. The cord blood plasma was removed, placed into 2 ml aliquots, and stored at -70°C until analysis. The buffy coat was harvested, diluted 1:1 with Iscove's modified Dulbecco's medium containing 2% fetal bovine serum (FBS) (Stem Cell Technologies, Vancouver, BC, Canada), then layered onto Ficoll-Paque PLUS^® ^and centrifuged for 30 min at 400 × *g *at room temperature. The light-density, mononuclear cell (MNC) layer was harvested and washed twice in Iscove's modified Dulbecco's medium + 2% FBS, then washed once in PBS containing 2% FBS. Another complete blood count with automated differential was then performed to determine the MNC count.

### Flow cytometric analysis of hematopoietic stem cell populations

We measured the currently recognized, standard populations of hematopoietic progenitor and stem cells (that is, the CD34^+ ^cells and CD34^+^CD38^- ^cells) by flow cytometry; in addition, the CD34^+^c-kit^+ ^stem/progenitor population was also quantitated by this method. Umbilical cord-derived MNC (see above) were adjusted to a concentration of 1 × 10^6 ^cells in 50 μl PBS containing 2% FBS and were incubated with the following fluorochrome-conjugated antibodies (all from BD Biosciences Pharmingen, San Diego, CA, USA) for 30 minutes on ice, in the dark: anti-CD34-fluorescein isothiocyanate (FITC), anti-CD38-phycoerythrin, anti-c-*kit*-phycoerythrin, or the combination of anti-CD34-FITC and anti-CD38-phycoerythrin, or the combination of anti-CD34-FITC and anti-c-*kit*-phycoerythrin. Samples treated with mouse IgG-phycoerythrin and IgG-FITC, or no antibody, served as controls.

The treated cells were washed once with PBS, centrifuged, and then resuspended in 50 μl of 4% paraformaldehyde. After an overnight incubation at 4°C in the dark, the cell suspension was brought up to 400 μl in PBS and was analyzed using a multi-laser flow cytometer (FACSCalibur™; BD Biosciences Immunocytometry Systems, San Jose, CA, USA) at the University of Massachusetts Medical School Flow Cytometry Core Facility. Quality control of the flow cytometric analyses was performed on the BD Biosciences FACSCalibur™ on a daily basis. Particles of known fluorescent characteristics (Calbrite particles; BD Biosciences) were utilized to examine instrument alignment and sensitivity. Instrument daily quality control values were maintained and stored by the Flow Cytometry Core Facility staff. The CD34^+^, CD34^+^CD38^-^, and CD34^+^c-*kit*^+ ^hematopoietic progenitor populations were quantitated from the gated MNC population (lymphocytes and monocytes, based on forward versus side light scatter) using the FlowJo™ software program (Tree Star, Inc., Ashland, OR, USA); backgating demonstrated that all stem/progenitor subpopulations were within the gated MNC population. These progenitor populations were then normalized to 10^3 ^MNC counts and the values used in statistical analyses.

These three independently assayed stem/progenitor subpopulations were determined to be highly correlated to one another: Spearman correlation coefficients relating CD34^+^/10^3 ^MNC levels to CD34^+^CD38^-^/10^3 ^MNC levels and CD34^+^c-*kit*^+^/10^3 ^MNC levels were 0.86 (*P *< 0.0001) and 0.93 (*P *< 0.0001), respectively, and the Spearman correlation coefficient relating CD34^+^CD38^-^/10^3 ^MNC levels to CD34^+^c-*kit*^+^/10^3 ^MNC levels was 0.88 (*P *< 0.0001).

### Analysis of hematopoietic stem cell potential by GM-CFU assay

Primitive hematopoietic progenitor cells have the ability to form colonies *in vitro *in semisolid medium upon stimulation with specific growth factors. The growth of colonies derived from GM-CFU in semisolid medium characterizes the proliferative potential of stem cells [17] and is used here for verifying the results from flow cytometry. We have used GM-CFU colony formation in methylcellulose-based semisolid medium as a measurement of stem/progenitor populations within the cord blood samples. Cord blood MNC (2 × 10^4 ^cells) or CD34^+ ^cells (3 × 10^2 ^cells, isolated from the MNC population using the EasySep™ immunomagnetic cell enrichment system; Stem Cell Technologies) were plated in 35-mm tissue culture plates containing 1.1 ml methylcellulose medium (MethoCult™ GF H4534; Stem Cell Technologies). Plates were incubated at 37°C in a 95% air:5% CO_2 _humidified atmosphere and colonies consisting of 40 cells or greater were quantitated after 14 days using an Olympus model SZ-ILA stereo microscope. Based on a mean of GM-CFU counts from four plates, results were expressed as the number of GM-CFU/10^3 ^MNC. GM-CFU values were highly correlated to the FACS-based stem/progenitor measurements, with Spearman correlation coefficients relating GM-CFU/10^3 ^MNC values (*n *= 175) to CD34^+^/10^3 ^MNC, CD34^+^CD38^-^/10^3 ^MNC, and CD34^+^c-*kit*^+^/10^3 ^MNC of 0.88 (*P *< 0.0001), 0.76 (*P *< 0.0001), and 0.84 (*P *< 0.0001), respectively.

### Hormone assays

Hormone levels in the umbilical cord plasma samples were assayed at the ILAT Steroid RIA Laboratory, Department of Physiology, University of Massachusetts Medical School. Estradiol and testosterone were measured by radioimmunoassay using kits from Diagnostic Products Corporation (Los Angeles, CA, USA). The inter-assay coefficients of variation were 6.8% and 8.9%, and the intra-assay coefficients of variation were 3.4% and 5.6%, respectively. Unconjugated estriol, progesterone, prolactin, and SHBG were measured using chemiluminescent immunoassays from Diagnostic Products Corporation. The inter-assay coefficients of variation were 9.2%, 7.9%, 6.4%, and 4.8%, respectively, and the intra-assay coefficients of variation were 6.6%, 6.3%, 5.7%, and 2.0%, respectively. IGF-1 and IGFBP-3 were measured by immunoradiometric assay using IMMULITE (an automated method from Diagnostic Products Corporation), with inter-assay coefficients of variation of 9.0% and 8.0%, respectively, and intra-assay coefficients of variation of 3.3% and 4.8%, respectively. The quality control coefficients of variation were calculated using internal control samples included with each assay.

### Statistical analysis

Descriptive statistics on the characteristics of study population and laboratory data were calculated. Spearman rank correlation coefficients were estimated for bivariate analyses. Multivariate linear regression was used to examine the association between hormones (independent variable) and natural log-transformed measures of stem cell potential (dependent variable), adjusting for maternal and neonatal characteristics (mother's age, race of parents, number of live births, gestation duration, baby's gender and birth weight, delivery time, and study site). Maternal age, gestational duration, and birth weight were treated as continuous variables. The fitted coefficients from the regression analyses were exponentiated to obtain the estimated proportional change in outcome associated with each independent variable. Statistical significance was set at 0.05 (two-sided). To conduct statistical analyses, the SAS program (version 9.1; SAS Institute, Cary, NC, USA) was used.

## Results

Maternal and newborn characteristics, cord blood hormone levels, and cord blood cell populations of the total samples (*n *= 289) are presented in Table 1. With the exception of ethnicity, the maternal and newborn characteristics, including mother's age and education levels, gestation period, time and method of delivery, and infant's birth weight, were similar between the Worcester-derived samples and the Boston-derived samples (data not shown). The Boston cord blood samples had higher GM-CFU concentrations (5.4 ± 3.2 colonies/10^3 ^MNC) than the previously reported Worcester samples (0.84 ± 0.47 colonies/10^3 ^MNC), but this was due to a methodological change in which cells were plated in a more efficacious commercial growth medium for obtaining GM-CFUs.

**Table 1:** Summary of the maternal and newborn characteristics, cord blood hormone levels, and cord blood cell populations

Variable	*n*	Mean^a^	Range
Subject characteristics			
Mother's age (years)	289	30.0 ± 5.5	18.0–43.0
Race/ethnicity of mother and biological father			
Both Caucasian		147 (58.33%)	
Both African-American		20 (7.94%)	
Both Asian		39 (15.48%)	
Both Hispanic		15 (5.95%)	
Mixed		31 (12.30%)	
No data		37	
Parity			
First		118 (43.54%)	
Second		77 (28.41%)	
Third		46 (16.97%)	
Fourth and above		30 (11.07%)	
No data	18		
Prepregnancy weight (kg)	199	62.7 ± 12.3	38.3–112.5
Gestation duration (weeks)	289	39.6 ± 1.2	36.5–44.0
Gender of the baby			
Male		134 (50.57%)	
Female		131 (49.43%)	
No data	24		
Birth weight (g)	288	3,402.4 ± 431.9	2,256–4,660
Cord blood volume (ml)	289	92.5 ± 24.6	44.5–201.5
Cord blood plasma hormone levels			
Estradiol (ng/dl)	289	930.9 ± 528.6	40.6–3,305.4
Unconjugated estriol (ng/ml)	289	331.0 ± 141.4	18.1–1,459.5
Testosterone (ng/ml)	289	1.7 ± 0.9	0.3–9.2
Sex hormone-binding globulin (nmol/l)	289	22.8 ± 8.0	6.7–55.0
Progesterone (ng/ml)	289	226.6 ± 134.4	26.5–841.0
Prolactin (ng/ml)	289	254.0 ± 103.6	47.4–686.0
Insulin-like growth factor-1 (ng/ml)	289	81.2 ± 52.5	10.2–306.7
Insulin-like growth factor binding protein-3 (ng/ml)	289	941.1 ± 309.9	300.0–2,534.0
Cord blood cell populations			
Initial total nucleated cells × 10^6^/ml	288^b^	16.1 ± 5.2	6.3–40.6
Initial mononuclear cells × 10^6^/ml	288^b^	7.6 ± 2.8	2.8–20.8
CD34^+ ^cells/10^3 ^mononuclear cells	289	8.9 ± 5.5	0.4–35.5
CD34^+^CD38^- ^cells/10^3 ^mononuclear cells	289	4.1 ± 2.7	0.0–14.4
CD34^+^c-*kit*^+ ^cells/10^3 ^mononuclear cells^c^	249	7.6 ± 4.9	0.2–34.4
Granulocyte–macrophage colony-forming units/10^3 ^mononuclear cells^d^	175	5.4 ± 3.2	0.5–19.7

^a^Data presented as mean ± standard deviation or mean (%). ^b^Initial total nucleated cells/ml and mononuclear cells/ml values not determined in one sample. ^c^Only samples from the Tufts–New England Medical Center were assayed. ^d^Data only from Tufts–New England Medical Center-derived samples, due to a change in assay method.

The correlation of the cord blood plasma levels of the eight-factor panel of sex hormones, growth factors, and their associated binding proteins with measurements of stem cell potential of the umbilical cord samples (that is, CD34^+^, CD34^+^CD38^-^, and CD34^+^c-*kit*^+ ^pools and GM-CFU values, all expressed per 10^3 ^cord blood MNC) was then assessed by bivariate analysis. As shown in Table 2, Spearman correlation coefficients between IGF-1 cord blood plasma levels and all four quantitations of stem and progenitor populations were highly significant (coefficients ranging from 0.18 to 0.21). The correlation of IGFBP-3 with these stem/progenitor populations was less strong, with significant correlations only between this protein level and the CD34^+ ^and CD34^+^c-*kit*^+ ^subpopulations. Cord blood plasma estriol levels correlated significantly with the concentrations of the more primitive hematopoietic stem cell pools (that is, the CD34^+^CD38^- ^and CD34^+^c-*kit*^+ ^populations). Interestingly, the cord plasma levels of the SHBG, representing a potential negative regulator of sex hormone action, were found to be negatively correlated to each measurement of stem/progenitor potential (Spearman correlation coefficients of -0.12 to -0.16).

**Table 2:** Spearman correlation coefficients (*P *values) between cord blood sex hormones, growth factors, and associated binding proteins and cord blood hematopoietic progenitor/stem cell populations

Hormone/binding protein	Total nucleated cells/ml	Mononuclear cells/ml	Measures of stem cell potential, counts/10^3 ^mononuclear cells			
			
			CD34^+ ^(*n *= 289)	CD34^+^CD38^- ^(*n *= 289)	CD34^+^c-*kit*^+ ^(*n *= 249)^a^	Granulocyte–macrophage colony-forming units (*n *= 175)^b^
Estradiol	0.14 (0.02)	0.15 (0.01)	0.05 (0.37)	0.02 (0.79)	0.06 (0.36)	0.03 (0.67)
Estriol	0.20 (0.0006)	0.15 (0.01)	0.09 (0.14)	0.14 (0.02)	0.15 (0.02)	0.11 (0.16)
Testosterone	0.19 (0.001)	0.13 (0.03)	-0.03 (0.60)	0.01 (0.80)	0.02 (0.80)	0.06 (0.41)
Sex hormone-binding globulin	0.003 (0.96)	0.05 (0.36)	-0.12 (0.04)	-0.13 (0.03)	-0.13 (0.03)	-0.16 (0.04)
Progesterone	0.15 (0.009)	0.15 (0.01)	-0.01 (0.84)	-0.04 (0.50)	-0.008 (0.90)	-0.009 (0.91)
Prolactin	0.27 (<0.0001)	0.21 (0.0004)	-0.005 (0.93)	0.02 (0.70)	0.09 (0.17)	0.01 (0.90)
Insulin-like growth factor-1	-0.05 (0.41)	-0.09 (0.14)	0.18 (0.002)	0.19 (0.001)	0.21 (0.001)	0.18 (0.02)
Insulin-like growth factor binding protein-3	0.10 (0.10)	0.08 (0.20)	0.14 (0.01)	0.11 (0.07)	0.12 (0.05)	0.11 (0.14)

^a^Determined only in the Tufts–New England Medical Center-derived samples. ^b^Data only from the Tufts–New England Medical Center-derived samples.

Multiple linear regression analysis was then performed on these data to take into account potential confounding factors involving maternal and neonatal variables including mother's age, race of parents, number of live births, gestation duration, delivery time, birth weight, offspring gender, and study site (Table 3). IGF-1 levels remained strongly associated with all cord blood stem/progenitor concentrations, with each standard-deviation increase in IGF-1 being associated with a 15–19% increase in stem/progenitor concentrations. IGFBP-3 levels were again positively correlated to CD34^+ ^and CD34^+^c-*kit*^+ ^pools, and estriol levels to CD34^+^CD38^- ^and CD34^+^c-*kit*^+ ^pools, with a one standard-deviation increase in estriol being associated with an 11–12% increase in the concentration of these populations. By this analysis, SHBG remains negatively correlated to these stem/progenitor populations, but only the association with CD34^+ ^pools was statistically significant.

**Table 3:** Multiple linear regression analysis^a ^for the association between measurements of hematopoietic stem cell populations and umbilical cord blood plasma levels of hormones and associated binding proteins

Hormone/binding protein	CD34^+ ^cells/10^3 ^mononuclear cells		CD34^+ ^CD38^-^/10^3 ^mononuclear cells		CD34^+^c-*kit*^+^/10^3 ^mononuclear cells^b^		Granulocyte–macrophage colony-forming units/10^3 ^mononuclear cells^c^	
	
	% change^d ^(95% confidence interval)	*P *value	% change (95% confidence interval)	*P *value	% change (95% confidence interval)	*P *value	% change (95% confidence interval)	*P *value
Estradiol	3.8 (-4.6, 13.0)	0.39	3.0 (-6.1, 13.0)	0.52	6.6 (-4.4, 18.9)	0.25	2.6 (-8.9, 15.6)	0.67
Estriol	7.5 (-2.6, 18.6)	0.15	11.1 (-0.1, 23.6)	0.05	12.9 (0.8, 26.5)	0.04	10.4 (-3.0, 25.7)	0.13
Testosterone	0.2 (-7.4, 8.5)	0.95	2.1 (-6.3, 11.3)	0.63	2.3 (-6.3, 11.6)	0.62	0.2 (-8.4, 9.5)	0.97
Sex hormone-binding globulin	-7.9 (-15.3, 0.1)	0.05	-7.4 (-15.5, 1.5)	0.10	-8.3 (-17.0, 1.2)	0.09	-6.6 (-16.2, 4.1)	0.21
Progesterone	-0.2 (-8.3, 8.7)	0.97	-1.9 (-10.6, 7.6)	0.69	-0.9 (-9.9, 9.1)	0.86	0.8 (-10.1, 13.0)	0.90
Prolactin	-0.4 (-8.4, 8.4)	0.93	1.3 (-7.6, 11.0)	0.78	5.3 (-4.6, 16.1)	0.30	4.8 (-6.3, 17.2)	0.41
Insulin-like growth factor-1	17.3 (7.2, 28.4)	0.001	16.4 (5.5, 28.5)	0.003	19.2 (7.7, 32.0)	0.001	15.1 (2.6, 29.1)	0.02
Insulin-like growth factor binding protein-3	10.8 (1.3, 21.3)	0.03	8.1 (-2.1, 19.3)	0.12	10.9 (0.02, 23.0)	0.05	9.7 (-2.3, 23.0)	0.12

^a^From multiple regression model, adjusting for mother's age, race of parents (both Caucasian or not), number of live births, gestation duration, gender of baby, and delivery time (day or night). The study site (American Red Cross or Tufts–New England Medical Center) was also adjusted in the analysis of the pooled samples. ^b^Determined only in the Tufts–New England Medical Center-derived samples (*n *= 249). ^c^Data only from the Tufts–New England Medical Center-derived samples (*n *= 175). ^d^% change, expected proportional change in dependent variable associated with one standard-deviation increase per independent variable.

Different racial and ethnic groups in the United States have differing rates of breast cancer [18]. We explored whether the correlations between cord blood plasma levels of growth factors and hormones and concentration of hematopoietic stem cells would hold across various racial and ethnic groups. We divided the data by racial/ethnic categories, and bivariate analysis was repeated. As shown in Table 4, cord blood plasma levels of IGF-1 are positively and significantly correlated with all four measurements of stem/progenitor cell populations in the Caucasian subgroup, which represent the majority of samples (147 of 252 with known ethnicity). IGF-1 levels are also significantly correlated with some stem/progenitor measurements (CD34^+ ^and CD34^+^c-*kit*^+ ^pools) in the Hispanic-American subgroup (*n *= 15), but not in the African-American (*n *= 20), Asian-American (*n *= 39), or mixed (*n *= 31) subgroups.

**Table 4:** Spearman correlation coefficients (*P *values) between cord blood sex hormones, growth factors, and associated binding proteins and hematopoietic stem/progenitor cell populations among different racial/ethnic groups

Stem/progenitor cell measurement	Estradiol	Estriol	Testosterone	Sex hormone-binding globulin	Progesterone	Prolactin	Insulin-like growth factor-1	Insulin-like growth factor binding protein-3
Both parents Caucasian (*n *= 147)								
CD34^+^/10^3 ^MNC	0.12 (0.16)	0.16 (0.06)	-0.04 (0.59)	**-0.18 (0.03)**	-0.01 (0.87)	-0.05 (0.52)	**0.24 (0.003)**	0.12 (0.15)
CD34^+^CD38^-^/10^3 ^MNC	0.13 (0.12)	**0.25 (0.002)**	0.05 (0.53)	**-0.16 (0.05)**	-0.02 (0.80)	-0.02 (0.80)	**0.23 (0.004)**	0.05 (0.58)
CD34^+^c-*kit*^+^/10^3 ^MNC	**0.21 (0.03)**	**0.30 (0.001)**	0.05 (0.61)	-0.17 (0.07)	-0.001 (0.99)	0.10 (0.29)	**0.26 (0.005)**	0.09 (0.32)
GM-CFU/10^3 ^MNC	0.11 (0.32)	**0.24 (0.03)**	0.13 (0.24)	-0.16 (0.16)	-0.02 (0.88)	0.13 (0.25)	**0.24 (0.03)**	0.07 (0.56)
Both parents African-American (*n *= 20)								
CD34^+^/10^3 ^MNC	-0.25 (0.28)	-0.27 (0.24)	-0.22 (0.34)	-0.33 (0.16)	-0.14 (0.57)	-0.23 (0.32)	0.09 (0.71)	0.19 (0.41)
CD34^+^CD38^-^/10^3 ^MNC	-0.25 (0.28)	0.008 (0.97)	-0.08 (0.75)	-0.32 (0.18)	-0.10 (0.67)	-0.02 (0.93)	-0.003 (0.99)	0.86 (0.72)
CD34^+^c-*kit*^+^/10^3 ^MNC	-0.40 (0.09)	-0.38 (0.11)	-0.31 (0.19)	-0.42 (0.07)	-0.23 (0.34)	-0.34 (0.15)	0.12 (0.63)	0.30 (0.20)
GM-CFU/10^3 ^MNC	-0.39 (0.15)	-0.40 (0.14)	-0.25 (0.36)	**-0.52 (0.04)**	-0.42 (0.12)	-0.13 (0.63)	-0.02 (0.94)	0.35 (0.20)
Both parents Asian-American (*n *= 39)								
CD34^+^/10^3 ^MNC	0.14 (0.38)	0.09 (0.58)	0.22 (0.19)	0.08 (0.65)	0.15 (0.37)	0.19 (0.25)	0.15 (0.36)	0.06 (0.73)
CD34^+^CD38^-^/10^3 ^MNC	0.14 (0.93)	0.07 (0.66)	0.17 (0.29)	0.03 (0.86)	0.08 (0.62)	0.14 (0.40)	0.15 (0.35)	0.004 (0.98)
CD34^+^c-*kit*^+^/10^3 ^MNC	0.13 (0.42)	0.10 (0.53)	0.19 (0.24)	0.08 (0.64)	0.15 (0.35)	0.19 (0.26)	0.16 (0.35)	0.05 (0.75)
GM-CFU/10^3 ^MNC	0.26 (0.25)	0.22 (0.33)	0.24 (0.30)	0.26 (0.26)	0.24 (0.29)	0.09 (0.71)	0.31 (0.17)	0.06 (0.78)
Both parents Hispanic-American (*n *= 15)								
CD34^+^/10^3 ^MNC	0.45 (0.10)	-0.16 (0.58)	-0.38 (0.17)	-0.04 (0.90)	-0.06 (0.83)	0.14 (0.63)	**0.51 (0.05)**	0.01 (0.97)
CD34^+^CD38^-^/10^3 ^MNC	**0.60 (0.02)**	0.24 (0.40)	0.01 (0.96)	-0.21 (0.45)	0.09 (0.76)	0.14 (0.61)	0.38 (0.16)	-0.11 (0.70)
CD34^+^c-*kit*^+^/10^3 ^MNC	0.38 (0.19)	-0.05 (0.86)	-0.35 (0.22)	-0.19 (0.51)	0.16 (0.57)	0.23 (0.44)	**0.62 (0.02)**	0.07 (0.82)
GM-CFU/10^3 ^MNC	-0.01 (0.98)	-0.47 (0.24)	-0.49 (0.22)	-0.46 (0.26)	0.22 (0.61)	-0.25 (0.55)	0.25 (0.55)	-0.20 (0.63)
Parents of differing ethnicity/race (mixed) (*n *= 31)								
CD34^+^/10^3 ^MNC	-0.06 (0.74)	-0.14 (0.46)	-0.03 (0.85)	-0.07 (0.71)	0.009 (0.96)	0.21 (0.25)	0.03 (0.87)	0.05 (0.80)
CD34^+^CD38^-^/10^3 ^MNC	-0.19 (0.32)	-0.18 (0.32)	-0.08 (0.68)	-0.03 (0.86)	-0.05 (0.79)	0.33 (0.07)	0.08 (0.65)	0.25 (0.18)
CD34^+^c-*kit*^+^/10^3 ^MNC	0.03 (0.87)	0.005 (0.98)	-0.002 (0.99)	-0.08 (0.68)	0.004 (0.98)	0.19 (0.31)	-0.04 (0.83)	-0.02 (0.93)
GM-CFU/10^3 ^MNC	0.19 (0.38)	0.23 (0.27)	0.24 (0.27)	-0.20 (0.34)	0.20 (0.34)	0.01 (0.95)	0.11 (0.62)	0.08 (0.72)

Those correlation coefficients that are significant (*P *≤ 0.05) are in boldface. GM-CSF, granulocyte–macrophage colony-forming units; MNC, mononuclear cells.

Similarly, cord blood plasma estriol levels are positively correlated with stem cell pools in the Caucasian subgroup, but not in any of the other subgroups (Table 4). Estradiol levels are positively and significantly correlated only to the CD34^+^c-*kit*^+ ^in Caucasians and to the CD34^+^CD38^- ^in Hispanic-Americans, but not in other racial/ethnic groups. Finally, SHBG was negatively but significantly correlated with some of the stem/progenitor concentrations in the Caucasian samples but, for the most part, not in the other subgroups.

## Discussion

The stem cell burden theory provides a mechanistic framework for understanding a number of epidemiological findings, including that high birth weight can be linked with breast cancer risk [2] and risk in other cancer sites [3-5]; that levels of certain mitogens (for example, IGF-1) are linked with both birth weight [19] and breast cancer risk [20]; and, finally, that mammographic breast density (mammary gland area per unit breast area), a correlate of breast tissue cell number [21], prenatal and perinatal factors [22] and, speculatively, breast stem cell number and activity [10], is a strong predictor of breast cancer risk [23] and has been associated with both IGF-1 serum levels [24] and birth weight [22].

Ethical and practical considerations preclude a direct testing of this hypothesis (that is, establishing whether or not there is an association between prenatal mitogens and breast stem cell quantities) in humans. As an indirect approach, we have used measurements of hematopoietic stem cell and progenitor cell concentrations in readily accessible umbilical cord blood samples as surrogates for general stem cell activity or 'stem cell potential'. Although hematopoietic stem cells are capable of giving rise to a wide range of nonhematopoietic cells [9], there has been no report on a direct link with breast stem cells. Levels of hematopoietic stem cells were therefore used in this study as proxy indicators of stem cell burden to correlate with cord blood plasma levels of endocrine factors, which represent the *in utero *concentrations of these factors.

A pilot study indicated that cord blood plasma levels of IGF-1, its major binding protein IGFBP-3, and to a lesser degree estriol are positively correlated with hematopoietic stem/progenitor concentrations [16]. This finding provided the first evidence linking perinatal growth factor and hormone levels to stem cell potential. In the present large study (289 samples), we document more firmly that cord blood plasma levels of IGF-1 are strongly correlated to all cord blood stem and progenitor concentrations examined, and that IGFBP-3 and estriol are positively correlated to at least some of these stem/progenitor measurements.

The results with respect to IGF-1 are not surprising, as there is considerable evidence that IGF-1 is a positive effector of hematopoiesis. This evidence includes the following: adding IGF-1 to *in vitro *cultures of CD34^+ ^or CD34^+^CD38^- ^precursors augments their proliferation in response to standard colony-stimulating factors such as GM-CSF, granulocyte colony-stimulating factor and IL-3 [25], in part by acting as an antiapoptotic factor [26]; *in vivo*, IGF-1 or the physiologic inducer of IGF-1, growth hormone, can stimulate erythropoieisis, myelopoieisis and granulopoiesis in hypophysectomized or myelosuppressed rodent model systems [27,28]; and, thirdly, growth hormone administered to patients with growth hormone deficiency coelevates IGF-1 levels and erythroid and myeloid progenitors [29].

There is less evidence linking IGFBP-3 levels with hematopoiesis. Recently, however, recombinant IGFBP-3 was shown to directly stimulate the proliferation of human CD34^+^CD38^- ^hematopoietic precursors in the absence of IGF-1 [30]. Furthermore, IGFBP-3 is one of the factors elaborated by embryonic stromal feeder-layer cells that can support hematopoietic stem cell growth [31]. Why estriol levels correlate with cord blood hematopoietic stem/progenitor concentrations is less clear, although estrogens have been used in conjunction with other growth factors to stimulate the *ex vivo *proliferation of erythroid precursors from avian bone marrow [32] and human cord blood [33].

The more relevant question in terms of the stem cell burden theory of breast cancer risk is whether IGF-1 and/or estrogen levels correlate with breast stem cell pool expansion. Although this question cannot be directly answered, it is apparent that both IGF-1 and estrogens are critical to the primitive mammary epithelium during development. In mouse knockout models, IGF-1 null mice have a sharply decreased formation of mammary terminal end buds and ductal branching [34], as do estrogen receptor alpha (ERα) null mice [35]. In sexually immature female rats that have been hypophysectomized (to eliminate the endogenous growth hormone/IGF-1 axis) and oophorectomized (to remove the major endogenous source of estrogens), treatment with IGF-1 and estradiol were synergistic for restoring full mammary gland development [36]; similar results were obtained in IGF-1 null mice [37]. It is reasonable to assume that a factor such as IGF-1 is acting on primitive breast stem and progenitor cells to drive these developmental changes.

Whether or not estrogens act directly on breast stem cells, however, is less clear. At present it is not known whether the most primitive breast stem cells are ERα^- ^and give rise to more committed ERα^+ ^cells, or *vice versa*. Murine mammary stem cells lack ERα, progesterone receptor, and erbB2 [38]. This pattern is the same as that found in the so-called 'basal' type of breast cancer, a subclass of breast cancer. Based on microarray gene expression analysis, this subclass of breast cancer possesses a relatively undifferentiated, stem-like expression profile [39]. In addition, cell culture methods for isolating and expanding breast epithelial precursor cells often do not utilize estrogen. On the other hand, human mammary epithelial cells with stem-like properties isolated from adult tissues are ERα^+ ^[40], and long-lived, slow cycling, ERα^+ ^cells, have been described in mouse mammary tissue [41]. Alternatively, a population of ERα^+ ^cells in the developing mammary gland might serve a paracrine function for adjacent ERα^- ^stem/progenitors. This would explain the synergy of estrogens and IGF-1 in mammary development even if breast stem cells lack ERα [42].

Finally, our data suggest that race/ethnicity may modify the correlations between *in utero*/perinatal levels of factors such as IGF-1 and estrogens and stem cell values. The associations between cord blood plasma levels of IGF-1, IGFBP-3, or estrogens and cord blood hematopoietic stem cell values were stronger in the Caucasian and Hispanic subgroups and were weaker or not present in the Asian-American and African-American subgroups. Comparatively low sample numbers for the latter three subgroups preclude making a definitive conclusion on this issue. Caucasians tended to rank high among the groups in terms of hematopoietic stem cell values, whereas Hispanic-Americans ranked low, but again these differences among the subgroups were not significant (data not shown). Other investigators have reported that cord blood from Caucasians contain relatively high hematopoietic stem cell concentrations among racial/ethnic groups [43]. Also, since the Asian-American group had the highest levels of estriol (data not shown), it is possible that the nonsignificant relations reflect the lowered study power to document associations within populations at the high or low ends of the hormone levels. Factors underlying these racial/ethnic differences in signaling molecule/stem cell correlations and breast cancer risk itself will require further study.

## Conclusion

Cord blood levels of IGF-1 are strongly positively associated with concentrations of all the cord blood stem and progenitor cells examined. IGFBP-3 and estriol levels are positively associated with at least some of these cell populations, whereas SHBG tends to show inverse associations with these populations. Our data therefore support the hypothesis that intrauterine levels of breast epithelial mitogens modulate stem/progenitor pools and could, thus, affect long-term risk of breast and perhaps other forms of cancer.

## Abbreviations

ERα = estrogen receptor alpha; FITC = fluorescein isothiocyanate; FBS = fetal bovine serum; GM-CSF = granulocyte–macrophage colony-forming units; IGF-1 = insulin-like growth factor-1; IGFBP-3 = insulin-like growth factor binding protein-3; MNC = mononuclear cell; PBS = phosphate-buffered saline; SHBG = sex hormone-binding globulin.

## Competing interests

The authors declare that they have no competing interests.

## Authors' contributions

TMS oversaw the laboratory research protocols and drafted the manuscript. WCS participated in the design of study protocol and collected patient samples and questionnaire information. HPL participated in the stem cell experiments and conducted analyses on flow cytometry data. QL participated in the design of the study and performed the statistical analysis. IB participated in the design of the questionnaire and coordinated the study. WO oversaw the hormonal assays. DPC participated in the design of clinical protocol and subject enrollment. PL participated in the analyses and interpretation of the results. PQ advised on the stem cell experiments and participated in the interpretation of results. KLN oversaw the clinical research protocols. C-CH conceived the study, participated in the design, analysis, and result interpretation. All authors read and approved the final manuscript.
